# The genome sequence of the Brown-spot Pinion, *Agrochola litura *(Linnaeus, 1761)

**DOI:** 10.12688/wellcomeopenres.20347.1

**Published:** 2023-11-10

**Authors:** David C. Lees, Natural History Museum Genome Acquisition Lab, Darwin Tree of Life Barcoding collective, Wellcome Sanger Institute Tree of Life programme, Wellcome Sanger Institute Scientific Operations: DNA Pipelines collective, Tree of Life Core Informatics collective, Darwin Tree of Life Consortium

**Affiliations:** 1 Natural History Museum, London, England, UK

**Keywords:** Agrochola litura, Brown-spot Pinion, genome sequence, chromosomal, Lepidoptera

## Abstract

We present a genome assembly from an individual female *Agrochola litura* (the Brown-spot Pinion; Arthropoda; Insecta; Lepidoptera; Noctuidae). The genome sequence is 772.2 megabases in span. Most of the assembly is scaffolded into 32 chromosomal pseudomolecules, including the W and Z sex chromosomes. The mitochondrial genome has also been assembled and is 15.55 kilobases in length. Gene annotation of this assembly on Ensembl identified 19,500 protein coding genes.

## Species taxonomy

Eukaryota; Metazoa; Eumetazoa; Bilateria; Protostomia; Ecdysozoa; Panarthropoda; Arthropoda; Mandibulata; Pancrustacea; Hexapoda; Insecta; Dicondylia; Pterygota; Neoptera; Endopterygota; Amphiesmenoptera; Lepidoptera; Glossata; Neolepidoptera; Heteroneura; Ditrysia; Obtectomera; Noctuoidea; Noctuidae; Xyleninae; *Agrochola*; *Agrochola litura* (Linnaeus, 1761) (NCBI:txid987869).

## Background

The Brown-spot Pinion, *Agrochola litura* (Linnaeus, 1761), otherwise classified in the genus *Anchoscelis* Guenée, 1839 (type species: *Noctua nitida* ([Denis & Schiffermüller], 1775), is a moth in the family Noctuidae, subfamily Xyleninae.

With a wingspan of 32–39 mm (forewing length 14–17 mm) ( Bretherton *et al.*, 1983; Waring *et al.*, 2017), adults usually have brownish chestnut-coloured forewings, greyer towards the base, with five black marks or dashes evenly spaced along the costa, and a brown stain between the stigmata and the costa.

*Agrochola litura* occurs throughout Britain and the Scilly and Channel Islands but not Ireland (apart from two records in County Down) ( Waring *et al.*, 2017), and in Europe from Southern Scandinavia southwest to the Pyrenees and the northern edge of the Mediterranean, in Turkey, and in Western Asia as far east as the Caspian Sea ( GBIF Secretariat, 2023).

In Britain the Brown-spot Pinion is found in a wide range of habitats including broad-leaved woodland and scrubland, hedgerows, parks, grasslands, heaths, fenland, and gardens ( Waring *et al.*, 2017).

The species is monovoltine, the adult flying in the Autumn (late August to October), relatively later in the South of Britain ( Randle *et al.*, 2019). It can be found feeding on overripe blackberries and ivy blossum. The species overwinters as an egg. In a study of noctuid moth fecundity in S. Bohemia ( Spitzer *et al.*, 1984), a female had a relatively low number of eggs (201), as well as a relatively low potential population growth rate compared to some migrant species. The larvae hatch in the Spring and are polyphagous on various herbaceous plants and grasses, later sometimes climbing onto trees such as *Salix*, *Crataegus* and *Quercus* to complete their growth ( Bretherton *et al.*, 1983). The greenish to pinkish brown larva ( Henwood *et al.*, 2020) makes a subterranean cocoon, usually in early June, and pupates within six weeks ( Waring *et al.*, 2017).

The species has been classed as vulnerable, with an annual decrease of 3.9%, a dramatic 82% decline in abundance overall, over 35 years in a study of Rothamsted trap catches ( Conrad *et al.*, 2006). Randle *et al*. (2019) characterised the Great Britain abundance decline between 1970 and 2016 of 73% as substantial, and distribution range has also declined in this period.

DNA barcode records on BOLD (as of 19/10/2023) comprise a single Barcode Index Number or BIN (BOLD: AAC8167). The nearest species on BOLD appears to be *Agrochola luteogrisea* (Warren, 1911) (BIN, BOLD:AFE5283) which is around 4.76% divergent and which it closely resembles. This genome could help clarify phylogenetic relationships in this group of moths, and several other genomes of *Agrochola* (s.l.) are available (e.g. *A. circellaris*, *A. lota*, *A. macilenta* ( Boyes *et al.*, 2023a, Boyes *et al.*, 2023b; Lees *et al.*, 2023)).

## Genome sequence report

**Figure 1.**
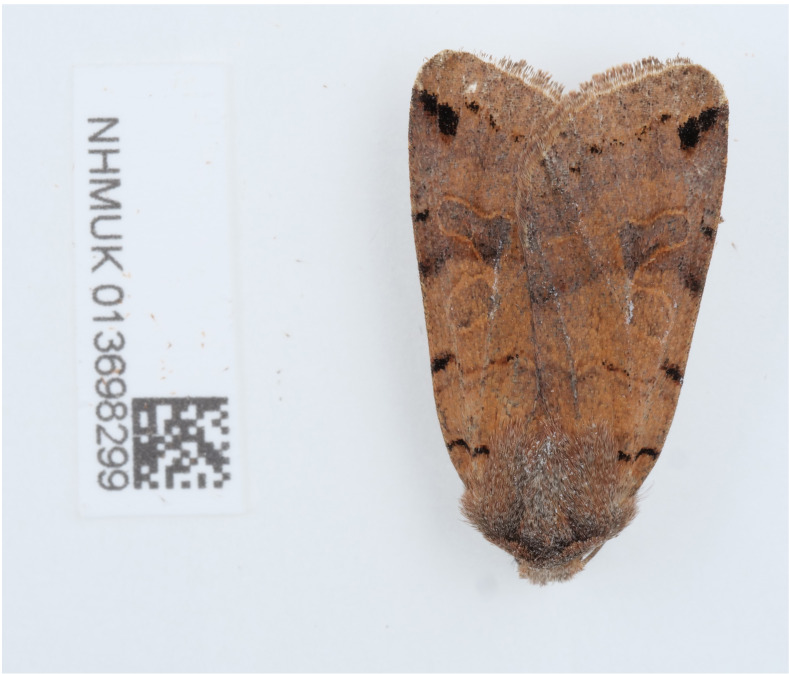
Photograph of the *Agrochola litura* (ilAgrLitu1) specimen used for genome sequencing.

The genome was sequenced from one female *Agrochola litura* ( Figure 1) collected from Restharrow Dunes National Nature Reserve, England. A total of 33-fold coverage in Pacific Biosciences single-molecule HiFi long reads was generated. Primary assembly contigs were scaffolded with chromosome conformation Hi-C data. Manual assembly curation corrected 36 missing joins or mis-joins and removed 6 haplotypic duplications, reducing the scaffold number by 13.24%.

**Figure 2.**
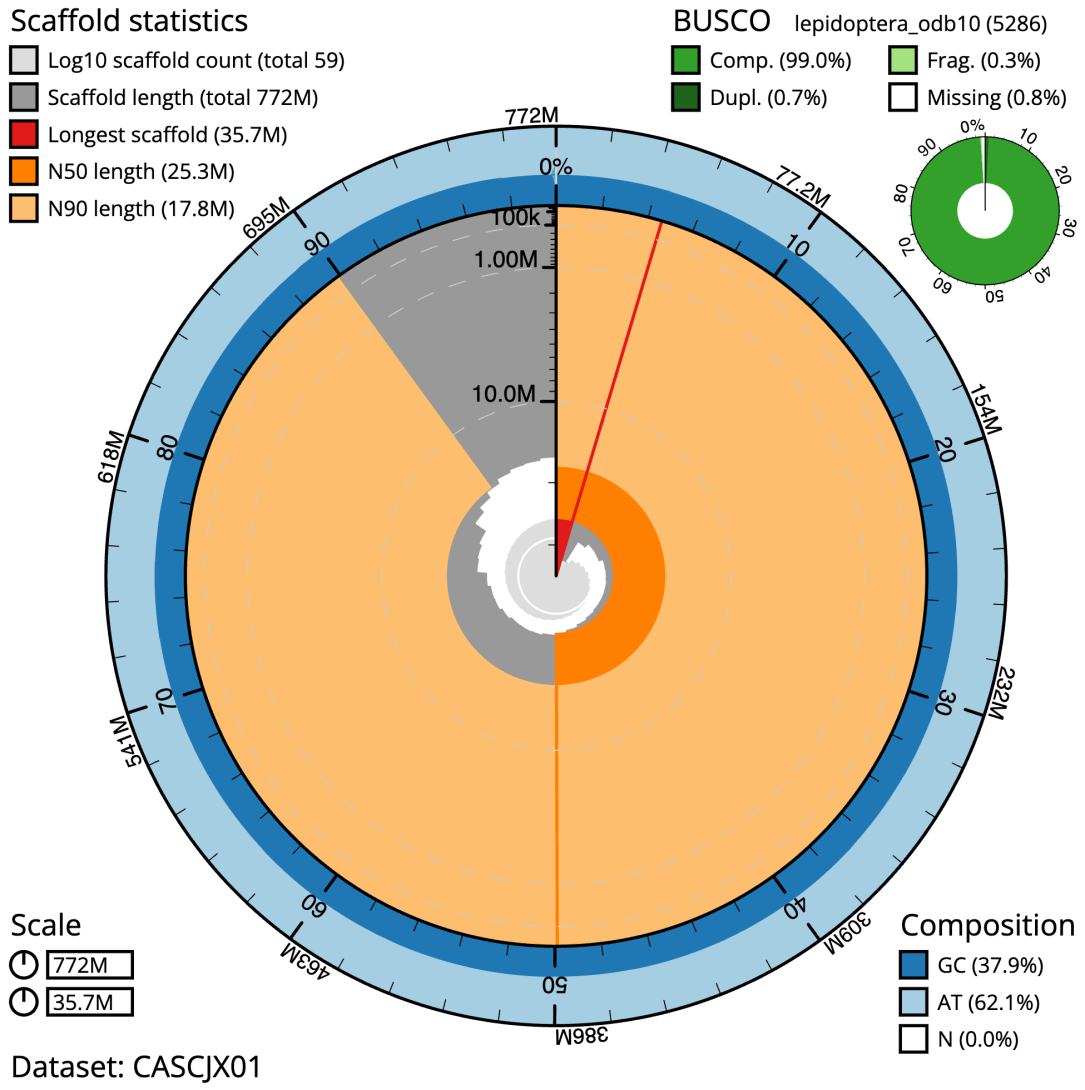
Genome assembly of *Agrochola litura*, ilAgrLitu1.1: metrics. The BlobToolKit Snailplot shows N50 metrics and BUSCO gene completeness. The main plot is divided into 1,000 size-ordered bins around the circumference with each bin representing 0.1% of the 772,191,765 bp assembly. The distribution of scaffold lengths is shown in dark grey with the plot radius scaled to the longest scaffold present in the assembly (35,726,591 bp, shown in red). Orange and pale-orange arcs show the N50 and N90 scaffold lengths (25,335,912 and 17,777,047 bp), respectively. The pale grey spiral shows the cumulative scaffold count on a log scale with white scale lines showing successive orders of magnitude. The blue and pale-blue area around the outside of the plot shows the distribution of GC, AT and N percentages in the same bins as the inner plot. A summary of complete, fragmented, duplicated and missing BUSCO genes in the lepidoptera_odb10 set is shown in the top right. An interactive version of this figure is available at https://blobtoolkit.genomehubs.org/view/Agrochola%20litura/dataset/CASCJX01/snail.

**Figure 3.**
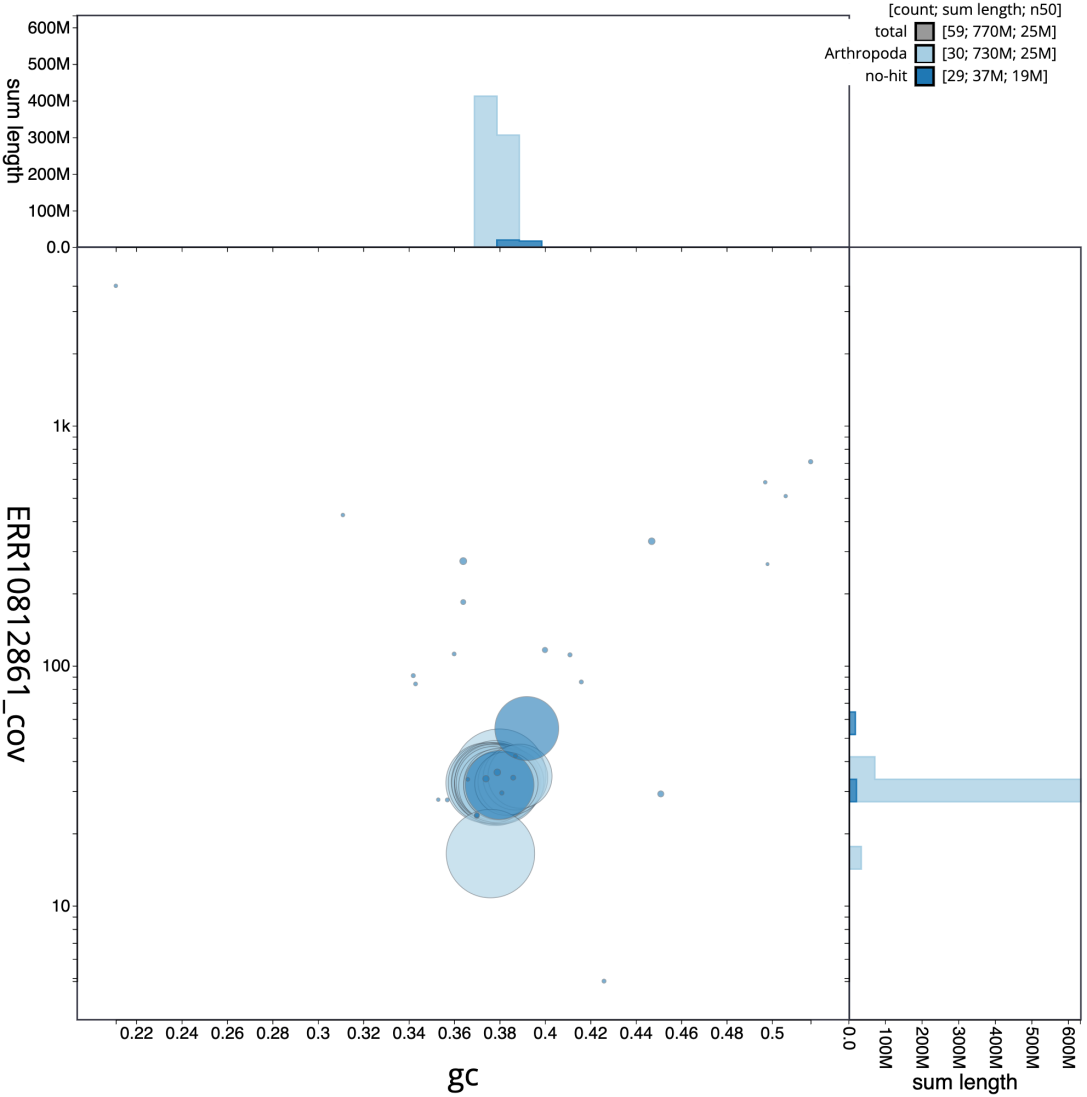
Genome assembly of *Agrochola litura*, ilAgrLitu1.1: BlobToolKit GC-coverage plot. Scaffolds are coloured by phylum. Circles are sized in proportion to scaffold length. Histograms show the distribution of scaffold length sum along each axis. An interactive version of this figure is available at https://blobtoolkit.genomehubs.org/view/Agrochola%20litura/dataset/CASCJX01/blob.

**Figure 4.**
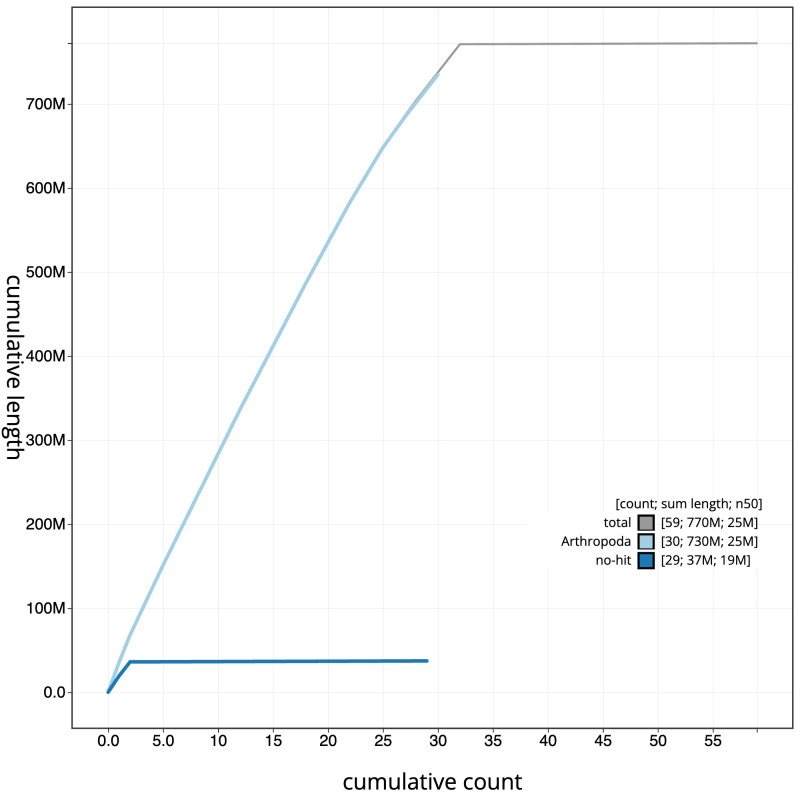
Genome assembly of *Agrochola litura*, ilAgrLitu1.1: BlobToolKit cumulative sequence plot. The grey line shows cumulative length for all scaffolds. Coloured lines show cumulative lengths of scaffolds assigned to each phylum using the buscogenes taxrule. An interactive version of this figure is available at https://blobtoolkit.genomehubs.org/view/Agrochola%20litura/dataset/CASCJX01/cumulative.

**Figure 5.**
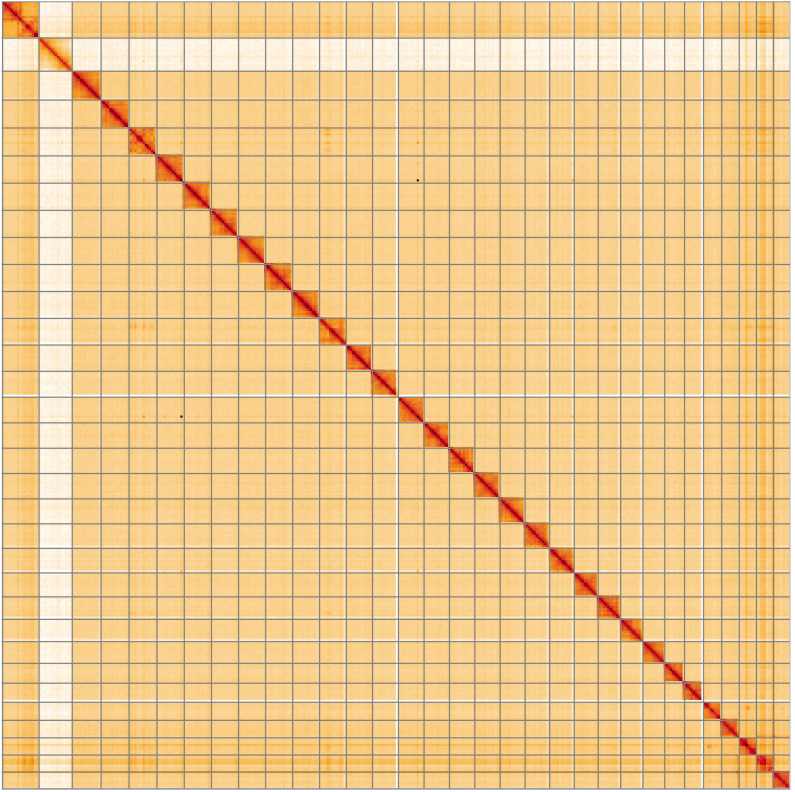
Genome assembly of *Agrochola litura*, ilAgrLitu1.1: Hi-C contact map of the ilAgrLitu1.1 assembly, visualised using HiGlass. Chromosomes are shown in order of size from left to right and top to bottom. An interactive version of this figure may be viewed at https://genome-note-higlass.tol.sanger.ac.uk/l/?d=awQT5bdURuSuMjttcRBwEw.

The final assembly has a total length of 772.2 Mb in 58 sequence scaffolds with a scaffold N50 of 25.3 Mb ( Table 1). The snailplot in Figure 2 provides a summary of the assembly statistics, while the distribution of assembly scaffolds on GC proportion and coverage is shown in Figure 3. The cumulative assembly plot in Figure 4 shows curves for subsets of scaffolds assigned to different phyla. Most (99.86%) of the assembly sequence was assigned to 32 chromosomal-level scaffolds, representing 30 autosomes and the Z and W sex chromosomes. Chromosome-scale scaffolds confirmed by the Hi-C data are named in order of size ( Figure 5; Table 2). While not fully phased, the assembly deposited is of one haplotype. Contigs corresponding to the second haplotype have also been deposited. The mitochondrial genome was also assembled and can be found as a contig within the multifasta file of the genome submission.

**Table 1.** Genome data for *Agrochola litura*, ilAgrLitu1.1.

Project accession data		
Assembly identifier	ilAgrLitu1.1	
Assembly release date	2023-03-11	
Species	*Agrochola litura*	
Specimen	ilAgrLitu1	
NCBI taxonomy ID	987869	
BioProject	PRJEB59302	
BioSample ID	SAMEA111458571	
Isolate information	ilAgrLitu1, female: head and thorax (DNA sequencing and Hi-C data), abdomen (RNA sequencing)	
Assembly metrics *		*Benchmark*
Consensus quality (QV)	66	*≥ 50*
*k*-mer completeness	100%	*≥ 95%*
BUSCO **	C:99.0%[S:98.2%,D:0.7%],F:0.3%,M:0.8%,n:5,286	*C ≥ 95%*
Percentage of assembly mapped to chromosomes	99.86%	*≥ 95%*
Sex chromosomes	Z and W chromosomes	*localised homologous pairs*
Organelles	Mitochondrial genome assembled	*complete single alleles*
Raw data accessions		
PacificBiosciences SEQUEL II	ERR10812861	
Hi-C Illumina	ERR10818323	
PolyA RNA-Seq Illumina	ERR12035178	
Genome assembly		
Assembly accession	GCA_949152395.1	
*Accession of alternate haplotype*	GCA_949152425.1	
Span (Mb)	772.2	
Number of contigs	161	
Contig N50 length (Mb)	9.4	
Number of scaffolds	58	
Scaffold N50 length (Mb)	25.3	
Longest scaffold (Mb)	35.7	
**Genome annotation**		
Number of protein-coding genes	19,500	
Number of gene transcripts	19,682	

* Assembly metric benchmarks are adapted from column VGP-2020 of “Table 1: Proposed standards and metrics for defining genome assembly quality” from ( Rhie *et al.*, 2021). ** BUSCO scores based on the lepidoptera_odb10 BUSCO set using v5.3.2. C = complete [S = single copy, D = duplicated], F = fragmented, M = missing, n = number of orthologues in comparison. A full set of BUSCO scores is available at https://blobtoolkit.genomehubs.org/view/Agrochola%20litura/dataset/CASCJX01/busco.

**Table 2.** Chromosomal pseudomolecules in the genome assembly of *Agrochola litura*, ilAgrLitu1.

INSDC accession	Chromosome	Length (Mb)	GC%
OX424490.1	1	35.73	38.0
OX424492.1	2	28.21	38.0
OX424494.1	4	27.4	38.0
OX424493.1	3	27.4	38.0
OX424495.1	5	26.7	37.5
OX424496.1	6	26.63	37.5
OX424497.1	7	26.51	37.5
OX424498.1	8	26.49	38.0
OX424499.1	9	26.48	37.5
OX424500.1	10	26.32	38.0
OX424501.1	11	26.09	38.0
OX424502.1	12	25.74	38.0
OX424503.1	13	25.34	37.5
OX424504.1	14	25.11	38.0
OX424505.1	15	24.88	38.0
OX424506.1	16	24.81	37.5
OX424507.1	17	24.78	38.0
OX424508.1	18	24.44	37.5
OX424509.1	19	24.07	37.5
OX424510.1	20	23.99	37.5
OX424511.1	21	23.49	38.0
OX424512.1	22	21.99	37.5
OX424513.1	23	21.78	38.0
OX424514.1	24	21.34	38.0
OX424515.1	25	19.44	38.0
OX424516.1	26	18.61	38.0
OX424517.1	27	17.78	38.5
OX424518.1	28	17.15	38.5
OX424519.1	29	16.81	39.0
OX424521.1	30	16.4	38.5
OX424520.1	W	16.69	39.0
OX424491.1	Z	32.47	37.5
OX424522.1	MT	0.02	21.0

The estimated Quality Value (QV) of the final assembly is 66 with *k*-mer completeness of 100%, and the assembly has a BUSCO v5.3.2 completeness of 99.0% (single = 98.2%, duplicated = 0.7%), using the lepidoptera_odb10 reference set ( *n* = 5,286).

Metadata for specimens, barcode results, spectra estimates, sequencing runs, contaminants and pre-curation assembly statistics are given at https://links.tol.sanger.ac.uk/species/987869.

## Genome annotation report

The *Agrochola litura* genome assembly (GCA_949152395.1) was annotated using the Ensembl rapid annotation pipeline ( Table 1; https://rapid.ensembl.org/Agrochola_litura_GCA_949152395.1/Info/Index). The resulting annotation includes 19,682 transcribed mRNAs from $PCG protein-coding and $NCG non-coding genes.

## Methods

### Sample acquisition and nucleic acid extraction

A female *Agrochola litura* (specimen ID NHMUK013698299, ToLID ilAgrLitu1) was collected from Restharrow Dunes National Nature Reserve, Sandwich Bay, England, UK (latitude 51.27, longitude 1.38) on 2021-09-24. The specimen was collected and identified by David Lees (Natural History Museum) and dry frozen at –80 °C.

High molecular weight (HMW) DNA was extracted at the Tree of Life laboratory, Wellcome Sanger Institute (WSI), using the main processes: sample preparation; sample homogenisation; HMW DNA extraction; HMW DNA fragmentation; and fragmented DNA clean-up. The ilAgrLitu1 sample was weighed and dissected on dry ice with tissue set aside for Hi-C sequencing (as per the protocol at https://dx.doi.org/10.17504/protocols.io.x54v9prmqg3e/v1). For sample homogenisation, tissue from the head and thorax was disrupted using a Nippi Powermasher fitted with a BioMasher pestle ( https://dx.doi.org/10.17504/protocols.io.5qpvo3r19v4o/v1). DNA was extracted at the Wellcome Sanger Institute (WSI) Scientific Operations core by means of the HMW DNA Extraction: Automated MagAttract protocol ( https://dx.doi.org/10.17504/protocols.io.kxygx3y4dg8j/v1). HMW DNA was sheared into an average fragment size of 12–20 kb in a Megaruptor 3 system with speed setting 30, following the protocol HMW DNA Fragmentation: Diagenode Megaruptor®3 for PacBio HiFi ( https://dx.doi.org/10.17504/protocols.io.8epv5x2zjg1b/v1). Sheared DNA was purified following either the Manual solid-phase reversible immobilisation (SPRI) protocol ( https://dx.doi.org/10.17504/protocols.io.kxygx3y1dg8j/v1), or the Automated SPRI protocol for ( https://dx.doi.org/10.17504/protocols.io.q26g7p1wkgwz/v1) for higher throughput. In brief, the method employs a 1.8X ratio of AMPure PB beads to sample to eliminate shorter fragments and concentrate the DNA. The concentration of the sheared and purified DNA was assessed using a Nanodrop spectrophotometer and Qubit Fluorometer and Qubit dsDNA High Sensitivity Assay kit. Fragment size distribution was evaluated by running the sample on the FemtoPulse system.

RNA was also extracted from abdomen tissue of ilAgrLitu1 in the Tree of Life Laboratory at the WSI using TRIzol, as per the protocol ( https://dx.doi.org/10.17504/protocols.io.yxmvm334nl3p/v1). RNA was then eluted in 50 μl RNAse-free water and its concentration assessed using a Nanodrop spectrophotometer and Qubit Fluorometer using the Qubit RNA Broad-Range (BR) Assay kit. Analysis of the integrity of the RNA was done using the Agilent RNA 6000 Pico Kit and Eukaryotic Total RNA assay.

### Sequencing

Pacific Biosciences HiFi circular consensus DNA sequencing libraries were constructed according to the manufacturers’ instructions. Poly(A) RNA-Seq libraries were constructed using the NEB Ultra II RNA Library Prep kit. DNA and RNA sequencing was performed by the Scientific Operations core at the WSI on Pacific Biosciences SEQUEL II (HiFi) and Illumina NovaSeq 6000 (RNA-Seq) instruments. Hi-C data were also generated from remaining head and thorax tissue of ilAgrLitu1 using the Arima2 kit and sequenced on the Illumina NovaSeq 6000 instrument.

### Genome assembly, curation and evaluation

Assembly was carried out with Hifiasm ( Cheng *et al.*, 2021) and haplotypic duplication was identified and removed with purge_dups ( Guan *et al.*, 2020). The assembly was then scaffolded with Hi-C data ( Rao *et al.*, 2014) using YaHS ( Zhou *et al.*, 2023). The assembly was checked for contamination and corrected as described previously ( Howe *et al.*, 2021). Manual curation was performed using HiGlass ( Kerpedjiev *et al.*, 2018) and Pretext ( Harry, 2022). The mitochondrial genome was assembled using MitoHiFi ( Uliano-Silva *et al.*, 2023), which runs MitoFinder ( Allio *et al.*, 2020) or MITOS ( Bernt *et al.*, 2013) and uses these annotations to select the final mitochondrial contig and to ensure the general quality of the sequence.

A Hi-C map for the final assembly was produced using bwa-mem2 ( Vasimuddin *et al.*, 2019) in the Cooler file format ( Abdennur & Mirny, 2020). To assess the assembly metrics, the *k*-mer completeness and QV consensus quality values were calculated in Merqury ( Rhie *et al.*, 2020). This work was done using Nextflow ( Di Tommaso *et al.*, 2017) DSL2 pipelines “sanger-tol/readmapping” ( Surana *et al.*, 2023a) and “sanger-tol/genomenote” ( Surana *et al.*, 2023b). The genome was analysed within the BlobToolKit environment ( Challis *et al.*, 2020) and BUSCO scores ( Manni *et al.*, 2021; Simão *et al.*, 2015) were calculated.

Table 3 contains a list of relevant software tool versions and sources.

**Table 3.** Software tools: versions and sources.

Software tool	Version	Source
BlobToolKit	4.1.7	https://github.com/blobtoolkit/blobtoolkit
BUSCO	5.3.2	https://gitlab.com/ezlab/busco
Hifiasm	0.16.1-r375	https://github.com/chhylp123/hifiasm
HiGlass	1.11.6	https://github.com/higlass/higlass
Merqury	MerquryFK	https://github.com/thegenemyers/MERQURY.FK
MitoHiFi	2	https://github.com/marcelauliano/MitoHiFi
PretextView	0.2	https://github.com/wtsi-hpag/PretextView
purge_dups	1.2.3	https://github.com/dfguan/purge_dups
sanger-tol/genomenote	v1.0	https://github.com/sanger-tol/genomenote
sanger-tol/readmapping	1.1.0	https://github.com/sanger-tol/readmapping/tree/1.1.0
YaHS	1.2a	https://github.com/c-zhou/yahs

### Genome annotation

The BRAKER2 pipeline ( Brůna *et al.*, 2021) was used in the default protein mode to generate annotation for the *Agrochola litura* assembly (GCA_949152395.1) in Ensembl Rapid Release.

### Wellcome Sanger Institute – Legal and Governance

The materials that have contributed to this genome note have been supplied by a Darwin Tree of Life Partner. The submission of materials by a Darwin Tree of Life Partner is subject to the **‘Darwin Tree of Life Project Sampling Code of Practice’**, which can be found in full on the Darwin Tree of Life website here. By agreeing with and signing up to the Sampling Code of Practice, the Darwin Tree of Life Partner agrees they will meet the legal and ethical requirements and standards set out within this document in respect of all samples acquired for, and supplied to, the Darwin Tree of Life Project.

Further, the Wellcome Sanger Institute employs a process whereby due diligence is carried out proportionate to the nature of the materials themselves, and the circumstances under which they have been/are to be collected and provided for use. The purpose of this is to address and mitigate any potential legal and/or ethical implications of receipt and use of the materials as part of the research project, and to ensure that in doing so we align with best practice wherever possible. The overarching areas of consideration are:

•   Ethical review of provenance and sourcing of the material

•   Legality of collection, transfer and use (national and international)

Each transfer of samples is further undertaken according to a Research Collaboration Agreement or Material Transfer Agreement entered into by the Darwin Tree of Life Partner, Genome Research Limited (operating as the Wellcome Sanger Institute), and in some circumstances other Darwin Tree of Life collaborators.

## Data Availability

European Nucleotide Archive: *Agrochola litura*. Accession number PRJEB59302; https://identifiers.org/ena.embl/PRJEB59302 ( Wellcome Sanger Institute, 2023). The genome sequence is released openly for reuse. The *Agrochola litura* genome sequencing initiative is part of the Darwin Tree of Life (DToL) project. All raw sequence data and the assembly have been deposited in INSDC databases. Raw data and assembly accession identifiers are reported in Table 1.

## Author information

Members of the Natural History Museum Genome Acquisition Lab are listed here: https://doi.org/10.5281/zenodo.4790042.

Members of the Darwin Tree of Life Barcoding collective are listed here: https://doi.org/10.5281/zenodo.4893703.

Members of the Wellcome Sanger Institute Tree of Life programme are listed here: https://doi.org/10.5281/zenodo.4783585.

Members of Wellcome Sanger Institute Scientific Operations: DNA Pipelines collective are listed here: https://doi.org/10.5281/zenodo.4790455.

Members of the Tree of Life Core Informatics collective are listed here: https://doi.org/10.5281/zenodo.5013541.

Members of the Darwin Tree of Life Consortium are listed here: https://doi.org/10.5281/zenodo.4783558.
