# Supplementary material for: The genome sequence of the Brown-spot Pinion, Agrochola litura (Linnaeus, 1761)
Source: Wellcome Open Res. 2023 Nov 10;8:512. [Version 1] doi: 10.12688/wellcomeopenres.20347.1 (PMC11128041; doi:10.12688/wellcomeopenres.20347.1)
Supplement: Peer review history

# Reviewer report: Reviewer response for version 1

**DOI:** 10.21956/wellcomeopenres.22531.r73775

**Published:** 2024-05-25

**Authors:** Muzafar Riyaz

**Affiliations:** 1 Xavier Research Foundation, St Xavier's College, Palayamkottai, Tamil Nadu, India

The paper provides valuable insights into the genomic characterization of *A. litura*, which could contribute to further research on phylogenetic relationships and conservation efforts within this moth group.

 Yes, the paper presents a clear rationale for creating the dataset by highlighting the importance of sequencing the genome of *Agrochola litura* for understanding phylogenetic relationships and aiding conservation efforts. The protocols employed for sample acquisition, nucleic acid extraction, sequencing, genome assembly, and annotation are described meticulously, indicating technically sound work. Furthermore, the paper provides comprehensive details of methods and materials, facilitating replication by others. The datasets, including genome assembly metrics, annotation results, and accession numbers, are presented clearly within the paper and deposited in public databases, ensuring usability and accessibility for other researchers. Therefore, the study appears to be methodologically robust, with transparent documentation of procedures and accessible datasets, enhancing its reliability and potential for scientific advancement.

Are sufficient details of methods and materials provided to allow replication by others?

Yes

Is the rationale for creating the dataset(s) clearly described?

Yes

Are the datasets clearly presented in a useable and accessible format?

Yes

Are the protocols appropriate and is the work technically sound?

Yes

Reviewer Expertise:

Phylogenomics

I confirm that I have read this submission and believe that I have an appropriate level of expertise to confirm that it is of an acceptable scientific standard.

# Reviewer report: Reviewer response for version 1

**DOI:** 10.21956/wellcomeopenres.22531.r73774

**Published:** 2024-04-08

**Authors:** Simon H Martin

**Affiliations:** 1 Institute of Evolutionary Biology, University of Edinburgh, Edinburgh, UK

This report describes a chromosome-scale genome assembly for the moth Agrochola litura. The approaches used are state-of-the-art and the assembly produced is of high quality. The assembly was produced from a single individual Agrochola litura using pacBio HiFi sequencing, which is excellent for insect genome assembly. Hi-C chromatin conformation sequencing was also used for scaffolding. The resulting assembly is therefore of excellent contiguity and sequence accuracy. I have no major concerns about the essmebly or article, but one minor concern about methodology reporting.

 Minor points

 1. The report states that RNA-seq data was generated, but it does not mention in the Genome Annotation section whether the RNA-seq data was used to support annotation. This should be clarified in the text.

Are sufficient details of methods and materials provided to allow replication by others?

Yes

Is the rationale for creating the dataset(s) clearly described?

Yes

Are the datasets clearly presented in a useable and accessible format?

Yes

Are the protocols appropriate and is the work technically sound?

Yes

Reviewer Expertise:

Evolutionary genomics

I confirm that I have read this submission and believe that I have an appropriate level of expertise to confirm that it is of an acceptable scientific standard.
